# Comparative Efficacy and Safety of Daily Versus Alternate-Day Dosing of Rosuvastatin in Diabetic Dyslipidemia: An Open-Label Clinical Trial

**DOI:** 10.7759/cureus.85457

**Published:** 2025-06-06

**Authors:** Nalini Kumari, Chetna A Shamkuwar, Vijay M Motghare, Ganesh N Dakhale

**Affiliations:** 1 Department of Pharmacology, Apollo Institute of Medical Sciences and Research, Hyderabad, IND; 2 Department of Pharmacology, Government Medical College, Nagpur, IND; 3 Department of Pharmacology, All India Institute of Medical Sciences, Nagpur, IND

**Keywords:** alternate day regimen, ascvd, dyslipidemia, rosuvastatin, sea

## Abstract

Background: Cardiovascular disease (CVD) is a growing health concern among individuals with diabetes. Cardiovascular conditions tend to present earlier in Indian populations, and the prevalence of diabetes is increasing in the region. Type 2 diabetes (T2DM) is frequently linked to abnormal cholesterol (CH) levels, which can increase the risk of heart problems. Rosuvastatin is a medication often used to manage cholesterol and may offer dosing flexibility due to its long duration of action.

Aims and objectives: To compare the efficacy and safety of daily versus alternate-day dose of rosuvastatin in patients with T2DM.

Materials and methods: The study was a single-center, prospective, open-label, parallel-group, randomized trial conducted over 12 weeks. The study enrolled 60 patients with diabetes-related dyslipidemia who were receiving care at a diabetic clinic within a tertiary care teaching center. Patients were randomized into two groups: group I received a daily dose and group II received an alternate-day dose of rosuvastatin. The primary endpoint was the percentage change in low-density lipoprotein cholesterol (LDL-C). Secondary endpoints included percentage changes in total cholesterol (TC), triglycerides (TGs), high-density lipoprotein cholesterol (HDL-C) levels, and adverse events over 12 weeks of treatment. Appropriate tests were applied, considering a significance level of p<0.05. Paired t-tests were used for within-group comparisons, unpaired t-tests for between-group comparisons, and the Mann-Whitney test was applied to evaluate differences in the percentage changes in lipid profile parameters. The study adhered to the International Council for Harmonisation & Good Clinical Practice (ICH & GCP) guidelines. Approval from the Institutional Ethics Committee (IEC) and informed consent (IC) were obtained.

Results: There was reduction in serum TC, TG, LDL-C, and very-low-density lipoprotein (VLDL) levels and an increase in HDL-C levels in both groups. After 12 weeks, significant improvements in lipid parameters were observed in both treatment groups (p<0.05), with the daily dosing group showing slightly more significant improvements. There was a significant decrease in TC level (p<0.0001) in the daily dose regimen, while significant decrease in LDL-C level (p<0.0001) in the alternate-day regimen of rosuvastatin dosing. Common adverse events included dizziness, palpitations, myalgia, malaise, and uneasiness, with no serious adverse events reported.

Conclusion: Alternate-day rosuvastatin treatment demonstrated comparable efficacy and safety to a daily dosing regimen, with significant reductions in TC, TG, LDL-C, and VLDL levels and an increase in HDL-C level. The alternate-day regimen is economically advantageous and provides an alternative option for patients who are intolerant to daily statin therapy.

## Introduction

Diabetes is a chronic metabolic disorder characterized by elevated levels of blood glucose (or blood sugar), which, over time, can lead to serious damage to the heart, blood vessels, eyes, kidneys, and nerves. Type 1 diabetes was formerly known as juvenile or insulin-dependent diabetes mellitus (IDDM), while type 2 diabetes (T2DM) is a chronic condition in which the pancreas produces little to no insulin. The rates of diabetes and obesity are expected to continue rising in recent years [1]. Over the past two decades, the incidence of diabetes in Southeast Asia (SEA) has exceeded all previous predictions, with a steady increase observed worldwide. According to "The IDF Diabetes Atlas," approximately 537 million adults aged 20-79, constituting 10.5% of the global adult population in this age group, are affected. Projections suggest that this number will increase to 643 million by 2030 and 783 million by 2045. According to the "2021 United Nations World Population Report," the global prevalence of diabetes was estimated at 10.5%, ranging from 8.3% to 12.0%. The prevalence was higher among men (10.8%) than women (10.2%) [1].

"Approximately 240 million people worldwide are living with undiagnosed diabetes, meaning nearly one in two adults with the condition are unaware of it. Nearly 90% of those with undiagnosed diabetes reside in low- and middle-income countries, with the SEA and Western Pacific regions having the highest rates of undiagnosed cases as per the IDF data." Additionally, over 1.2 million children and adolescents have type 1 diabetes, with more than half (54%) being under the age of 15. A systematic review of the literature found that between 2006 and 2017, the incidence of diabetes remained stable in over 70% of predominantly high-income populations [2,3].

"The ICMR's study 'India Diabetes (INDIAB)', a large-scale nationwide survey, revealed significant variations in diabetes prevalence across countries like India" [4]. The study found notably higher rates of diabetes in urban areas and in states with higher per capita income, highlighting a connection between socioeconomic factors and diabetes prevalence. "These findings are based on data collected from 15 representative Indian states, covering 51% of the adult population [4,5]. Various comorbidities such as obesity and lipid abnormalities are linked to an increased risk of developing diabetes complications, as previous studies indicate that lipid abnormalities are prevalent in individuals with T2DM" [6-8]. In the current scenario, the prevalence of coronary heart disease (CHD) tends to occur nearly a decade earlier in India compared to Western countries. While CHD incidence has been declining in the West, deaths from CHD in middle-income countries like India have almost doubled over the past decade [9,10]. The main reasons for CHD are the rising cases of growing obesity, increased prevalence rate of T2DM, lifestyle changes related to food habits, a sedentary lifestyle, and a growing economy, which are the contributing factors to chronic diseases like diabetes. According to previous literature, T2DM significantly increases the risk of dyslipidemia and cardiovascular (CV) risk disease. Lipids and glucose are essential for energy metabolism. It is well known that diabetes is often associated with dyslipidemia, which is characterized by elevated triglyceride (TG), low levels of high-density lipoprotein cholesterol (HDL-C), elevated low-density lipoprotein cholesterol (LDL-C) particles, and higher concentrations of apolipoprotein-B (ApoB) containing particles [11].

In dyslipidemia, there is a significant and critical link between diabetes and CVD, and additionally, hyperglycemia further accelerates atheroma formation in patients with dyslipidemia, as shown in previous studies [12-14]. In 2020, approximately 4.51 million deaths occurred due to high LDL-C levels, marking a 19% increase since 2010, according to the "American Heart Association (AHA)." Between 2017 and 2020, about 32.8% of adult males and 36.2% of adult females in the United States had total cholesterol (TC) levels of 5.172 mmol/l (200 mg/dl) or higher. During the same period, LDL-C levels of 3.362 mmol/l (130 mg/dl) or higher were found in 25.6% of U.S. males and 25.4% of U.S. females.

Additionally, 24.9% of U.S. males and 9.3% of U.S. females reported HDL-C levels below 1.034 mmol/l (40 mg/dl). India is now synonymous with these data [15]. Hypertriglyceridemia, low HDL-C, and a predominance of small, dense LDL particles can be detected before the clinical diagnosis of T2DM has been made in insulin-resistant individuals, related to prediabetic individuals with average glucose concentrations, as supported by various studies. This review will support the diabetic dyslipidemia hypothesis, its potential role in developing T2DM, and the effects of individual lipid components on CVD. The causal relationship between LDL and atherosclerosis is well established but not for HDL, and recent study data also indicate a causal role of LDL and TG-rich lipoproteins in CV events [16-18]. The pathogenesis of dyslipidemia involving the liver is characterized by the overproduction of VLDL, which contributes to elevated serum TG levels.

Atherosclerotic cardiovascular disease (ASCVD) increases the risk of CHD in diabetic patients, and LDL is recognized is recognized as a key factor in the development of ASCVD and its major clinical sequelae. Evidence for the causal role of other Apo-B containing lipoproteins in ASCVD is emerging [19-21]. Numerous epidemiologic, clinical, and experimental studies have correlated the critical role of LDL-C and its oxidized form in driving atherosclerosis progression. As a result, lowering LDL-C levels is a widely adopted clinical strategy for treating and preventing CHD [22-25]. "The American Diabetes Association (ADA)" and the "National Cholesterol Education Program (NCEP) ATP III guidelines" advocate for aggressive management of dyslipidemia in patients with T2DM. While lowering elevated LDL-C levels remains the primary focus, abnormalities in HDL-C and TG levels should also be proactively addressed [25]. FDA has approved several statins, including atorvastatin, rosuvastatin, simvastatin, pravastatin, fluvastatin, lovastatin, and pitavastatin. For individuals at high risk of ASCVD, more intensive management strategies such as high-intensity statin therapy are recommended. Statins are the first-line treatment for elevated LDL-C levels [26]. Previous studies indicate that rosuvastatin, an HMG-CoA reductase inhibitor, is highly effective in lowering total and LDL-C levels, achieving greater LDL-C reduction than other statins. Notably, rosuvastatin at doses of 10 to 80 mg has been shown to lower LDL-C more significantly compared to atorvastatin at the same doses and other statins like simvastatin and pravastatin [27-29]. "'CRESTOR trial' demonstrated that rosuvastatin calcium significantly enhances lipid profiles in patients with various dyslipidemia conditions." It effectively lowers Total-C, LDL-C, TG, and Apo-B while increasing HDL-C in individuals with primary hypercholesterolemia (with or without hypertriglyceridemia), familial and non-familial hypercholesterolemia, mixed hyperlipidemia, and non-insulin-dependent diabetes mellitus (NIDDM).

The terminal half-life of rosuvastatin is approximately 18-20 hours, which may allow for alternate-day medication administration. However, dose-dependent adverse effects such as myalgia and elevated hepatic enzymes caused by statin therapy are similar to those caused by other statins, as shown in previous studies. Statins like rosuvastatin should be prescribed carefully in patients with predisposing factors for muscle pain and a rise in creatinine kinase; these features are more common in the elderly (aged 65 years or older), those with inadequately treated hypothyroidism, or those with renal impairment. A few cases of rhabdomyolysis have been reported earlier [30,31]. Additionally, the risk has been increased with specific other lipid-lowering therapies such as fibrates, niacin, and gemfibrozil. Other drugs, such as cyclosporine, lopinavir/ritonavir, or atazanavir/ritonavir, also increase the risk of myopathy. Furthermore, concomitant alcohol use and liver disease can also aggravate the risk of myopathy and muscle pain [32-34]. However, clinical trials have shown that patients tolerate rosuvastatin well during treatment.

## Materials and methods

The aim of this study was to evaluate and compare the efficacy and safety of daily versus alternate-day rosuvastatin administration in managing dyslipidemia among patients with T2DM.

This study was a single-center, randomized, prospective, open-label, parallel-group trial conducted over 12 weeks. The study was carried out in the diabetic OPD clinic of the Department of Medicine at a Tertiary Care Teaching Hospital. The primary endpoint was the percentage change in LDL-C levels from baseline to 12 weeks of treatment with either a daily or alternate-day dose of 10 mg rosuvastatin. Secondary endpoints included changes in TC, TG, HDL-C levels, and the incidence of any adverse events over the 12 weeks. Safety was assessed by measuring liver profiles like serum glutamic oxaloacetic transaminase (SGOT), serum glutamic pyruvic transaminase (SGPT), blood urea, and serum creatinine at the beginning and end of the study.

Participants who met the inclusion criteria were enrolled following the guidelines of the "NCEP" and "ATP III" [25,26]. The inclusion criteria were as follows: T2DM patients aged between 20 and 75 years who were on oral antidiabetic therapy (naive patients). Others to be included were patients having LDL-C levels >100 mg/dl and at least one of the following: TC levels >200 mg/dl, TG levels >150 mg/dl, HDL levels <40 mg/dl for men, or HDL levels <50 mg/dl for women. Both male and female patients were included to ensure better cohort representation. The exclusion criteria were as follows: patients with T1DM; patients with T2DM on insulin; those with significant CVD (e.g., history of CHF, CHD, or MI within the last year); those with clinically significant liver or kidney disorders; pregnant or lactating women; individuals who had used statins within the three months preceding the study; those on other lipid-lowering agents, oral contraceptives, corticosteroids, or antipsychotic medications; and patients with known hypersensitivity to the study medications. All participants were enrolled after meeting the inclusion and exclusion criteria during the screening process. Safety was assessed by monitoring the proportion of patients experiencing adverse effects by the end of the study. Adverse drug reaction was identified using the "WHO-UMC system for standardised case causality assessment.''

The study adhered to International Council for Harmonisation & Good Clinical Practice (ICH & GCP) guidelines and received approval from the IEC. Informed consent (IC) was obtained from all participants who agreed to participate in the study. Sixty patients were randomized using a computer-generated table into two groups: Group I received 10 mg of rosuvastatin daily and Group II received the same dose on alternate days for 12 weeks. The randomization was implemented to maintain the integrity of the study.

All laboratory tests were conducted at the Central Research Laboratory (CRL) in the Department of Clinical Biochemistry at the Tertiary Care Teaching Hospital. Descriptive statistics, including mean and standard deviations (SD), were used to analyze the data. Lipid profiles, liver function tests (LFTs), renal function tests (RFTs), and blood glucose levels were compared using paired t-tests for within-group comparisons before and after treatment and unpaired t-tests for between-group comparisons at baseline. The Mann-Whitney test was applied to evaluate differences in the percentage changes in lipid profile parameters between the two groups when data were not normally distributed. Statistical significance was determined at p<0.05, and all analyses were done using GraphPad Prism 6.0.

Statistical analysis

The sample size was initially calculated to be 50, based on an absolute precision of 10% and a 95% confidence level (α error of 5%). Accounting for a 10% drop-out rate and ensuring a study power of over 80% (1-β), a total of 60 patients were enrolled in the study. PS software was used to calculate the sample size.

## Results

**Figure 1:**
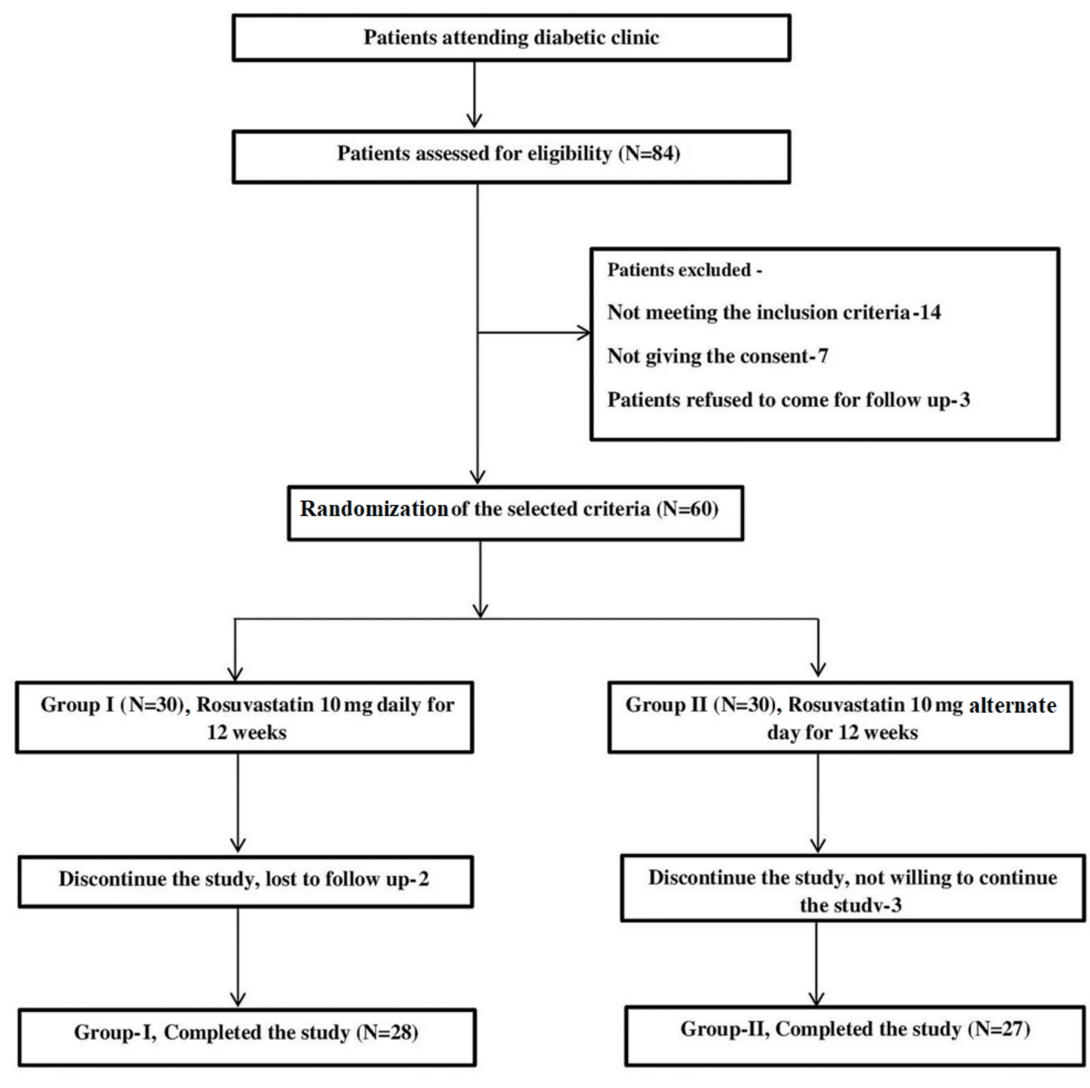
Flowchart showing the distribution of two groups.

The study enrolled 60 eligible patients, who were randomized into two groups: Group I received a daily dose of rosuvastatin and Group II received an alternate-day dose. The progression of participant recruitment and allocation is shown in Figure 1. A total of 28 patients in Group I and 27 patients in Group II completed the study. Two subjects were lost to follow-up in Group I and three subjects were not willing to continue in the study. For statistical analysis, only subjects who completed the study were considered.

Figure 1 shows the distribution of patients in two groups from screening of patients to the completion of the study.

Table 1 outlines the baseline characteristics of patients in both Group I and Group II. Both groups are comparable and show no statistically significant difference at baseline, and both groups had comparable lipid profiles (p>0.05).

**Table 1:** Baseline characteristics of patients in Group I and Group II. Values are expressed in mean ± SD, n= number of patients; p>0.05 (not significant). An unpaired t-test was used for intergroup comparisons, with a significance level of (p<0.05). BMI: body mass index; TC: total cholesterol; TG: triglyceride; LDL: low-density lipoprotein; HDL: high-density lipoprotein; VLDL: very low-density lipoprotein; SGOT: serum glutamic oxaloacetic transaminase; SGPT: serum glutamic pyruvic transaminase.

Characteristic	Group I (n=28) (rosuvastatin 10 mg daily)	Group II (n=27) (rosuvastatin 10 mg alternate day)	p-value
Patients	28	27	0.8075
Age in years (mean ± SD)	58.79 ± 9.678	58.74 ± 10.435	0.9868
Male	15	15	0.8075
Female	13	12	
BMI (mean ± SD)	23.645± 2.706	23.878 ± 2.295	0.7331
TC (mg/dl)	212.1 ± 6.987	209.7 ± 6.886	0.808
TG (mg/dl)	229.0 ± 23.72	185.2 ± 16.99	0.1415
LDL (mg/dl)	124.6 ± 6.550	133.556 ± 31.487	0.3213
VLDL (mg/dl)	45.89 ± 4.743	37.45 ± 3.217	0.1495
HDL (mg/dl)	41.43 ± 1.938	40.93 ± 2.049	0.8592
FBS (mg/dl)	125.0 ± 5.621	124.0 ± 6.374	0.8998
PPBS (mg/dl)	181.1 ± 8.390	183.6 ± 7.956	0.8284
SGOT (U/l)	27.96 ± 2.119	22.89 ± 1.625	0.0640
SGPT (U/l)	23.75 ± 2.322	19.30 ± 1.741	0.1329
Blood urea (mg/dl)	23.75 ± 1.208	23.78 ± 1.252	0.9873
Serum creatinine (mg/dl)	0.9211 ± 0.0377	0.8852 ± 0.0491	0.5632

Table 2 presents a comparison of lipid profiles between Group I (n=28) and Group II (n=27). There was a decrease in serum TC, TG, LDL-C, and VLDL levels and an increase in HDL-C levels in both groups. After 12 weeks, significant improvements in lipid parameters were observed in both treatment groups (p<0.05), with the daily dosing group showing slightly more significant improvements. There was a significant decrease in TC levels (p<0.0001) in the daily dose rosuvastatin regimen compared to the alternate-day regimen (p<0.0015), while LDL-C levels showed a significant decrease (p<0.0001) in the alternate-day rosuvastatin regimen compared to the daily dose regimen (p<0.0002).

**Table 2:** Comparison of lipid profile at baseline and at 12 weeks in both Group I and Group II. *p<0.05 (significant) used for fall or rise in lipid parameters. **p<0.001 (highly significant) for fall or rise in lipid parameters. A paired t-test was used for group comparison, with a significance level of p<0.05; Group I (rosuvastatin 10 mg daily regimen) and Group II (rosuvastatin 10 mg alternate-day regimen). TC: total cholesterol; TG: triglyceride; LDL: low-density lipoprotein; HDL: high-density lipoprotein; VLDL: very low-density lipoprotein.

Lipid parameters	Group I (mean ± SD)		p-value	Group II (mean ± SD)		p-value
	At baseline	At 12 weeks		At baseline	At 12 weeks	
TC (mg/dl)	212.071 ±36.974	175.171 ±22.544**	< 0.0001	209.674 ±35.781	188.659 ±17.745*	0.0015
TG (mg/dl)	229.036 ±125.502	152.371 ±63.821**	0.0003	185.222 ±88.264	149.444 ±54.841*	0.0067
LDL (mg/dl)	124.607 ±34.660	96.486 ±18.583**	0.0002	133.556 ± 31.487	109.519 ±19.526*	< 0.0001
HDL(mg/dl)	41.429 ±10.258	49.286 ±6.480**	0.0004	40.926 ±10.648	46.519 ±7.298*	0.0131
VLDL (mg/dl)	45.893 + 25.099	30.574 + 12.881	0.0003	37.452 ±16.714	29.185 ±10.408*	0.0014

Table 3 shows the percentage reduction in mean LDL-C levels at 12 weeks. The once-daily rosuvastatin 10 mg regimen resulted in a 22.57% reduction, while the alternate-day regimen showed a 17.99% reduction. The p-values for these reductions were p=0.0002 and p=0.0001, respectively. The daily dosing regimen provided an additional 4.58% reduction in LDL-C, as shown in Table 3. There was a significant reduction in TC (17.40% in Group I and 10.02% in Group 2) and TG (33.47% in Group I and 19.31% in Group II). Additionally, there was a significant increase in HDL, with 18.97% in daily regimen and 13.67% in alternate-day regimen in both Groups I and II, respectively. VLDL level was also reduced, showing a 33.38% in Group I and 22.07% in Group II.

**Table 3:** Mean percentage change of lipid parameter at 12 weeks in both Group I and Group II. ^*^The Mann-Whitney test was used for intergroup comparisons between Group I and Group II, with a significance level of p<0.05. ^†^Median and range were calculated for skewed data; Group I: rosuvastatin 10 mg daily regimen, Group II: rosuvastatin 10 mg alternate-day regimen. TC: total cholesterol; TG: triglyceride; LDL: low-density lipoprotein; HDL: high-density lipoprotein; VLDL: very low-density lipoprotein.

Lipid parameters	Mean percentage change ± SD from baseline		p-value*
	Group I	Group II	
TC (mg/dl)	-17.400 ± (-3.902)	-10.023 ± (-5.040)	0.5784
	-16.68 (-41.25 - 18.92)^†^	-11.40 (-31.93 - 36.50)^†^	
TG (mg/dl)	-33.473 ± (-4.914)	-19.316 ± (-3.786)	0.0561
	-33.87 (-63.68 - 54.62)^†^	-24.85 (-56.75 - 106.3)^†^	
LDL (mg/dl)	-22.568 ± (-4.638)	-17.998 ± (-3.798)	0.3358
	-24.57 (-53.70 - 40.51)^†^	-19.53 (-39.24 - 46.27)^†^	
HDL (mg/dl)	18.966 ± (-3.682)	13.665 ± (-3.146)	0.0899
	30.23 (-27.08 - 75.00)^†^	14.58 (-27.66 - 161.9)^†^	
VLDL (mg/dl)	-33.379 ± (-4.867)	-22.073 ± (-3.773)	0.1111
	-33.94 (-61.77 - 53.85)^†^	-25.37 (-56.75 - 106.3)^†^	

Table 4 shows the comparison of other laboratory parameters at baseline and at 12 weeks. Although there was an increase in serum SGOT/SGPT levels with rosuvastatin in both groups, none of the patients had levels of SGOT/SGPT more than three times the upper normal limits or more than five times the upper normal limits. No patients were dropped from the study due to any serious side effects. There were no significant changes in the levels of blood urea, serum creatinine, FBS, and PPBS. During the study, a total of five patients (17.86%) reported adverse effects in Group I, i.e., who received a daily regimen of rosuvastatin (10 mg). Dizziness, palpitation, myalgia, malaise, and uneasiness were the most commonly reported adverse events. All adverse effects were transient, and discontinuation of therapy was not required. No serious adverse events related to study of the drug were observed. 

**Table 4:** Comparison of other laboratory parameters at baseline and at 12 weeks in both Group I and Group II. None of the patient had more than three times upper normal levels of serum SGOT and SGPT during therapy. A paired-t test was used for group comparison, with a significance level of p<0.05. SGOT: serum glutamic oxaloacetic transaminase; SGPT: serum glutamic pyruvic transaminase; FBS: fasting blood sugar; PPBS: postprandial blood sugar.

Lipid parameters	Group I (mean ± SD)		p-value	Group II (mean ± SD)		p-value
	At baseline	At 12 weeks		At baseline	At 12 weeks	
SGOT	23.643 ± 10.386	27.964 ± 11.210	0.0375	22.889 ± 8.441	25.185 ± 7.270	0.0241
SGPT	23.750 ± 12.286	27.071 ± 14.013	0.0204	19.296 ± 9.046	22.037 ± 8.211	0.0481
Blood urea	23.750 ± 6.392	22.511 ± 6.474	0.2724	23.778 ± 6.506	23.815 ± 6.385	0.9750
Serum creatinine	0.921 ± 0.200	0.855 ± 0.123	0.0780	0.885 ± 0.255	0.852 ± 0.264	0.3063
FBS	125.036 ± 29.745	120.964 ±47.557	0.6372	123.963 ± 33.122	119.926 ± 26.617	0.4192
PPBS	181.107 ± 44.396	175.393 ±45.339	0.4075	183.630 ± 41.342	169.741 ± 37.426	0.1077

**Figure 2:**
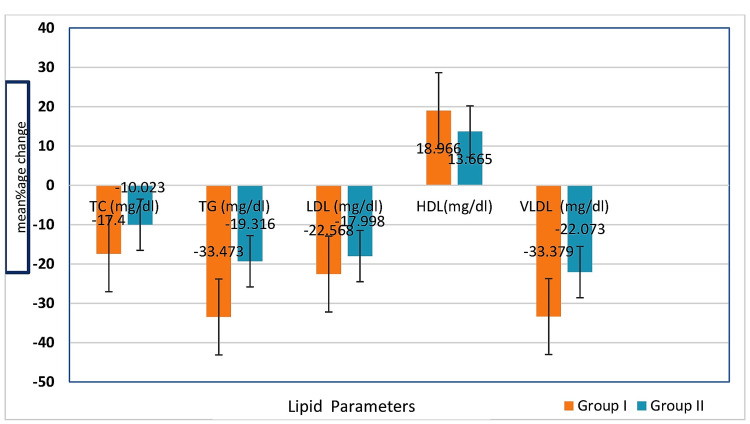
Mean percentage change of lipid profile at 12 weeks in both Group I and Group II. The Mann-Whitney test was used for intergroup comparisons, with a significance level of p<0.05 (for skew data); Group I: rosuvastatin 10 mg daily regimen, Group II: rosuvastatin 10 mg alternate-day regimen. TC: total cholesterol; TG: triglyceride; LDL: low-density lipoprotein; HDL: high-density lipoprotein; VLDL: very low-density lipoprotein.

Figure 2 shows the graphical representation of comparison of lipid profile in both groups (Group I and Group II); all details are also shown in Table 3. There was a decrease in serum TC, TG, LDL-C, HDL-C, and VLDL levels, and the percentage change in these levels was significantly decrease by 17.4%, 33.47%, 22.57%, and 33.38%, respectively. There was a significant increase of HDL-C level by 18.97% in Group I rosuvastatin 10 mg daily regimen and in Group II rosuvastatin 10 mg alternate-day regimen, the percentage change in these levels were significantly decrease by 10.02%, 19.31%, 17.99%, and 22.07%, respectively. There was a significant increase of HDL-C level by 13.67%.

## Discussion

Diabetic dyslipidemia can be treated, and its risk factors can be modified with lifestyle and diet, which is commonly seen in individuals with T2DM patients. Theories suggest that it accelerates the formation of atherosclerotic lesions, significantly raising the risk of CVD [35]. Statins are commonly prescribed to treat dyslipidemia in patients with T2DM, aiding beneficiaries in both primary and secondary prevention of CVD [36,37]. Rosuvastatin is highly effective in lowering elevated levels of LDL-C, TC, and TG while increasing HDL-C in patients with primary hypercholesterolemia, familial hypercholesterolemia, and mixed dyslipidemia, as supported by much existing research.

However, HDL-C and other lipoproteins also play crucial roles in the management of dyslipidemia [38]. Rosuvastatin is the most potent statin, achieving an LDL-C reduction of over 50% (55-60% at 40 mg), followed by atorvastatin (45-50% at 80 mg) and pitavastatin (45-50% at 4 mg) [39]. "Other research, such as a "study by Li J," reported even higher reductions in LDL-C (37%) with daily dosing, and the "STELLAR study" observed a 46-55% reduction with higher doses of rosuvastatin (10-40 mg)" [40,41]. The differences in LDL-C reduction across studies may be attributed to variations in patient demographics and regional factors. The alternate-day dosing regimen also showed significant improvements in LDL-C levels. For instance, Dafda et al. reported a 32.76% vs. 29.18% reduction in LDL-C with daily vs. alternate-day rosuvastatin dose 10 mg and Vasa et al. observed a 33.5% in the daily regimen and 31% in the alternate-day regimen rosuvastatin 10 mg [42,43].

"'The 2018 AHA/ACC/Multisociety Guideline on Blood Cholesterol Management,' the '2019 ESC/EAS Guidelines on Dyslipidemia Management', the '2021 ESC Guidelines on cardiovascular Disease Prevention', and the '2021 Canadian Society Guidelines on Dyslipidemia Management' all emphasize the causal role of LDL-C and Apo-B containing lipoproteins in the development of ASCVD'' [44]. An inverse relationship was found between HDL-C levels and the incidence of clinically significant atherosclerosis, primarily due to the reverse cholesterol transport (RCT) pathway and its role in reducing CVD risk [45,46]. In this study, HDL-C levels increased by 18.97% with daily rosuvastatin (10 mg) and 13.67% with alternate-day dosing. These results are consistent with the findings of Vasa et al. [43], which reported increases of 19.89% and 17.09% in daily and alternate-day regimens, respectively, at the end of the sixth week. Additionally, Panchavarthi et al. showed significant improvement (p<0.0001) in both groups [47].

Regarding the decrease in TC, the daily rosuvastatin regimen resulted in a 17.4% reduction from baseline over 12 weeks, which is comparable to the results of studies by Farnier et al. with a reduction of 10.3% and Panchavarthi et al. with a reduction of 24% [45,47]. The alternate-day regimen also showed a reduced TC level, although to a lesser extent, with a 10.02% decrease observed. Similar reductions were reported in studies by Juszczyk et al. with a reduction of (−46.4±7.9%) and Panchavarthi et al. showed 21.6% reduction, although no statistically significant difference was found between the two dosing regimens in this study [47,48]. Moderately elevated TG levels are a growing concern for CVD risk, particularly as they are often associated with metabolic syndrome, a cluster of conditions that includes insulin resistance, obesity, hypertension, low HDL-C levels, a procoagulant state, and a significantly increased risk of CVD. 'This study reduced TG levels by 33.47% with daily dosing and by 19.31% with alternate-day dosing over 12 weeks with "Thapa et al. (2017)," shows 25.6% reduction in with 5 mg rosuvastatin treatment for three months and these results also align with findings from "Vasa et al. (2023)," which reported reductions of 25.56% and 41.33% for daily and alternate-day regimens, respectively' [49]. All these studies support the reduction of TG levels with rosuvastatin treatment, as TG is also a risk factor for CVD.

In this study, VLDL levels decreased by 22.07% with alternate-day dosing and by 33.38% with daily dosing, with both regimens showing statistically significant reductions. Similar decreases were reported by "Panchavarthi et al. (2016)" for daily rosuvastatin (10 mg) regimens, with a decrease of 26.71% in the daily regimen and 26.52% in the alternate-day regimen for three months.

Regarding safety, previous studies have noted adverse effects of statins such as myopathy, elevated transaminases, and rare occurrences of rhabdomyolysis, peripheral neuropathy, memory loss, sleep disturbances, and erectile dysfunction. However, statins are well tolerated and among the most widely chosen drugs by physicians [33,34].

Muscle-related side effects are the most common reason for statin intolerance or discontinuation [50]. In this study, both dosing regimens of rosuvastatin (10 mg) were well-tolerated, with only a few patients reporting mild side effects such as headache, malaise, myalgia, palpitations, and uneasiness, all of which resolved over time. No significant liver or kidney function changes were observed in either group over the 12-week study period, and no serious adverse effects were reported. All adverse effect causality assessments were done using the "WHO-UMC system for standardised case causality assessment." These results findings are supported by studies conducted by Vasa et al. and Awad et al. in meta-analysis, related to adverse events, which concluded that alternate-day dosing of statins, particularly atorvastatin, and rosuvastatin, is as effective as daily dosing in reducing LDL-C, TG, and HDL-C levels [43,51,52].

Study limitations

This study was an open-label study, which may introduce potential biases. The absence of blinding in such a design can introduce various forms of bias, including performance bias, detection bias, and reporting bias. It may affect both participant behavior and outcome assessment, potentially compromising the internal validity and generalizability of the study findings. Large samples are more robust and less susceptible to the influence of outliers. In this study, CPK test was not performed due to cost factor, and adverse effects were analyzed using WHO-UMC causality assessment. CPK test might provide a better understanding to correlate with statin-induced myopathy. Cost analysis has not been conducted in this current study.

## Conclusions

Rosuvastatin has been shown to significantly reduce elevated levels of LDL-C, TC, and TG while concurrently increasing HDL-C in both groups of patients diagnosed with primary hypercholesterolemia and mixed dyslipidemia and those suffering from diabetic dyslipidemia. Due to its long half-life, the beneficial lipid-modifying effects of rosuvastatin are evident with both daily and alternate-day dosing regimens. Notably, the alternate-day regimen provides a clinically effective and economically advantageous alternative, especially for patients with diabetic dyslipidemia who experience adverse effects or intolerance with daily statin therapy. This enhances long-term adherence and therapeutic outcomes with statin treatments. The current study was conducted on statin-naive patients with diabetic dyslipidemia.
